# A mechanically based magneto-inductive transmitter with electrically modulated reluctance

**DOI:** 10.1371/journal.pone.0199934

**Published:** 2018-06-28

**Authors:** Nathan Strachen, John Booske, Nader Behdad

**Affiliations:** Department of Electrical and Computer Engineering, University of Wisconsin-Madison, Madison, Wisconsin, United States of America; Helmholtz-Zentrum Dresden-Rossendorf, GERMANY

## Abstract

Magneto-inductive (MI) communication is a viable technology for wireless communications in underwater and underground environments. In this paper, a new design for an MI transmitter is presented. Unlike conventional MI transmitters that utilize coiled loops or solenoids to generate magnetic fields, we demonstrate the feasibility and advantages of using a rotating permanent magnet. We also present and experimentally verify a modulation technique that does not involve changing the rotational speed of the magnet. By electrically changing the permeability of a surrounding shield, the fields from the rotating magnet are amplitude modulated. Our findings suggest that increased efficiency and bandwidth can be realized compared to conventional MI transmitters.

## Introduction

In the last couple of decades, magneto-inductive (MI) communication systems have received much interest for applications involving underground and underwater communications. MI communication utilizes coiled loops or solenoids that generate and receive low frequency quasi-static magnetic fields. Because MI communication uses low frequency quasi-static (non-propagating) electromagnetic fields, signal attenuation through water, rock, or soil is less than those of RF communications [1]. In addition, MI is much less affected by changes in the channel medium than conventional technologies such as RF, acoustic, and light based communications. This has to do with the fact that MI communication depends primarily on the magnetic permeability of the channel medium. Water, rock, and soil in general have permeabilities close to that of air [2]. This makes MI a very good choice for communicating in harsh environments where other technologies would be unreliable. MI communications in shallow coastal areas where conventional techniques would suffer from multi-path propagation and noise have been demonstrated [2].

There are many practical applications for MI communications. MI has been used to develop reliable wireless sensors for monitoring bridge scouringn [3]. Bridge scouring is the process by which water erodes the sand and rocks from the supporting pillars of a bridge, which can cause the bridge to collapse. In addition, much work has been done in making reliable underground MI communication systems [4] [5] [6]. Some of this work has been prompted due to mining disasters such as the Sago Mine disaster that occurred in 2006 in West Virginia. An underground explosion in a coal mine trapped twelve miners; killing all but one due to carbon monoxide poisoning. Had there been proper communication systems available, the outcome might have been different [7]. Lockheed Martin in response to such disasters developed an MI system for wireless communications with underground mine workers known as MagneLink [8]. In addition to low-frequency wireless, work has also gone into developing MI based underground, underwater, and indoor navigation for a variety of applications [2], [9]–[10].

Ongoing research continues on MI communication due to its many applications. In this paper, we focus on improving the transmitter. Improvements in range, bandwidth, efficiency, volume, and weight are all of interest when designing an MI transmitter. Conventionally, coiled loops or solenoids are used to generate a magnetic moment. Work has been done to optimize the design of coiled loops and solenoids for MI communications [11]. Very recently, however, interest has been shown in producing a magnetic moment with a spinning magnet [12]–[13]. Compact neodymium magnets harboring strong magnetic fields can be spun on low friction bearings for ultra-high efficiency generation of the carrier frequency. However, challenges in high efficiency modulation remain. Modulation by mechanically changing the rotational speed of the magnet has been proposed [14]. However, this method can result in low bandwidth and poor efficiency due to mechanical inertia of the magnet and high rotational speeds.

In this paper, a novel modulation technique is proposed that does not involve changing the rotational speed of the magnet. Instead, a magnetic shield surrounds the spinning magnet. By electrically changing the permeability of the surrounding shield, the fields from the rotating magnet can be amplitude modulated. Our experimental results indicate that high efficiency and bandwidth are possible. We first present an analysis of the magnetic dipole moment from a single rotating magnet. We demonstrate a significant reduction in the maximum linear dimension of the mechanical transmitter compared to those of MI transmitters found in the literature. Experimental confirmation of the fields from a single rotating magnet is also shown. An analysis of different proposed modulation techniques for mechanically based MI transmitters was done, showing the viability of our modulation concept. Experimental demonstration of our proposed modulation technique is presented. Based on our results, we compare the efficiency of our prototype with that of an MI transmitter found in the literature, and to a mechanically based modulation approach. Our findings suggest that greater efficiency can be obtained with a mechanically based MI transmitter. In addition, we suggest future work for improving the performance of mechanical MI transmitters.

## Theory

### Fields from a spinning magnet

The magnitude of the magnetic moment produced by a conventional MI transmitter is given by

$$\begin{array}{c} m_{loop}=\mu_{r,eff}\pi a^{2}I_{0} \end{array}$$ (1)

where *a* is the radius of the loop, *μ*_*r*,*eff*_ is the effective relative permeability of the core (a function of the material and geometry of the core), and *I*_0_ is the total current through the loop [2]. The magnitudes of the vector components of the magnetic field for a loop that is parallel to the *z* − *y* plane, are given by [15]:

$$\begin{array}{c} B_{r}=\frac{\mu_{0}m_{loop}}{2\pi r^{3}}\text{cos}\;\left(\phi \right)\;\text{sin}\;\left(\theta \right),\;B_{\theta }=\frac{\mu_{0}m_{loop}}{4\pi r^{3}}\text{cos}\;\left(\phi \right)\;\text{cos}\;\left(\theta \right),\;B_{\phi }=\frac{\mu_{0}m_{loop}}{4\pi r^{3}}\text{sin}\left(\phi \right) \end{array}$$ (2)

For a permanent magnet, the equivalent magnetic dipole moment can be determined by the superposition of all the infinitesimal moments that compose the volume. Because we are interested in the magnetic field at distances much greater than the dimensions of the magnet, we can consider each infinitesimal dipole of the magnet as being located at the same point in space. Assuming a uniform magnetization $\vec{M}$ (with magnitude *M*_0_), the effective magnetic moment can therefore be computed by:

$$\begin{array}{c} m_{magnet}=M_{0}V_{m} \end{array}$$ (3)

Where *V*_*m*_ is the volume of the magnet [16]. We conclude that the fields at far distances from a magnet uniformly magnetized (in a single direction), do not depend on the specific dimensions of the magnet, but only on its volume.

**Fig 1:**
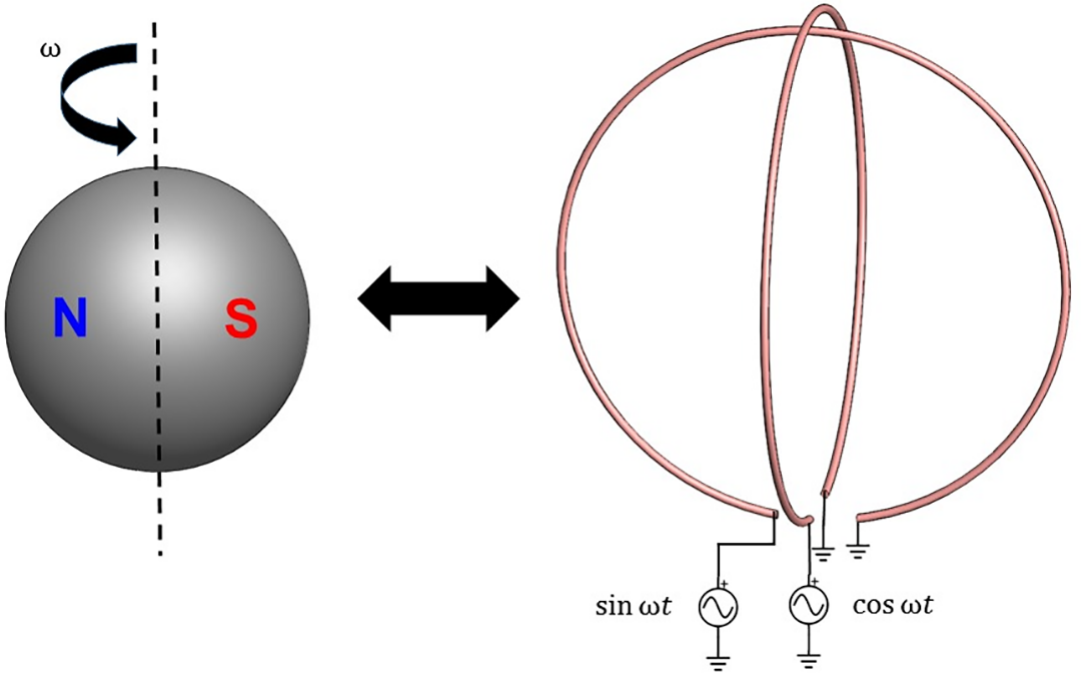
Modeling spinning magnet. Illustration of the electrical equivalent of a spinning magnet. Two orthogonal loops excited 90° out of phase produces a rotating magnetic dipole.

To produce an AC magnetic field, the given magnetic volume may be rotated. In order to produce the largest AC magnetic field value, the angular momentum of the magnet should be orthogonal to the magnetization direction. The rotating magnetic volume can be modeled by two orthogonal AC current loops, each with a magnetic moment given by (3), and excited 90° out of phase [17]. This is illustrated in Fig 1. The peak magnitude of the vector components of the magnetic field from a rotating magnet (with the angular momentum oriented in the *z* direction) are given by:

$$\begin{array}{c} B_{r}=\frac{\mu_{0}m_{magnet}}{2\pi r^{3}}\text{sin}\;\left(\theta \right),\;B_{\theta }=\frac{\mu_{0}m_{magnet}}{4\pi r^{3}}\text{cos}\;\left(\theta \right),\;B_{\phi }=\frac{\mu_{0}m_{magnet}}{4\pi r^{3}} \end{array}$$ (4)

We can use (1) and (3) to compare a rotating magnet with a conventional MI transmitter. Parameters that are of interest in building an MI transmitter are efficiency, volume, weight, and bandwidth. Efficiency is defined as the magnetic moment divided by the total input power of the transmitter [2]. To get a qualitative idea of what kind of advantage a mechanical transmitter could provide, we surveyed the different specifications of MI transmitters found in the literature. In Table 1, we show the maximum linear dimension of a coil or solenoid found in the literature, its magnetic moment, and frequency. Using (3), we also calculate the diameter of a magnetic sphere needed to produce the same magnetic moment. The sphere was assumed to be made from N52 neodymium magnet (with *M* = 1.17 × 10^6^ A/m). A significant reduction in the size of the transmitter can be observed.

**Table 1:** MI transmitters found in the literature. The maximum linear dimension is compared with the diameter of an N52 neodymium spherical magnet. The carrier frequency, magnetic moment, maximum linear dimension, and the magnet size for an equivalent magnetic moment are represented by *f*_*c*_, *m*_0_, *l*_*o*_, and *l*_*m*_ respectively.

Reference	Design	*f*_*c*_ [*Hz*]	*m*_0_ [*Am*^2^]	*l*_*o*_ [*cm*]	*l*_*m*_ [*cm*]
Markham [18]	Air Core Loop	2500	7.2	30	2.27
Sheinker [9]	Air Core Loop	630	800	100	10.9
Sojdehei [2]	Air Core Solenoid	630	110	56	5.64
Dinn [19]	Air Core Loop	575	30	130	3.68
Dinn [19]	Cored Solenoid	575	30	64	3.68

The efficiency of the mechanical transmitter depends on the efficiency of the motor that rotates the magnet and the power required to modulate the transmitter. If the magnet is supported by very low friction bearings, only a small amount of power would be required to sustain the rotation (assuming a constant rotational speed). The amount of power required to modulate the transmitter depends on the modulation technique.

### Modulation techniques

We envisioned four different possible modulation techniques. Out of all the proposed techniques, only mechanical frequency modulation has been proposed in the literature so far. We analyzed each one of the different techniques in terms of bandwidth and power efficiency. These techniques are described in the following paragraphs.

**Fig 2:**
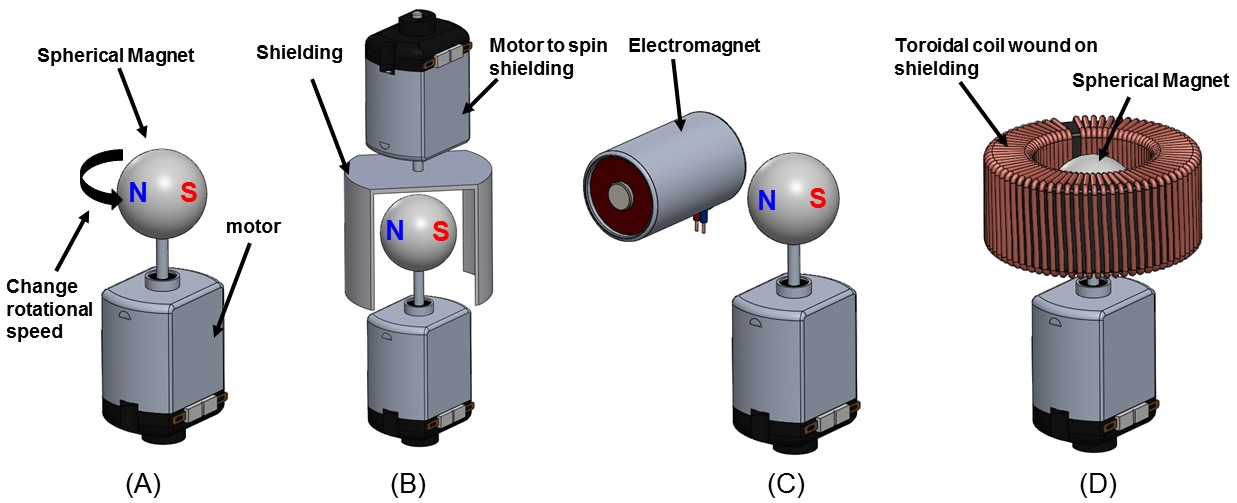
Modulation techniques. Illustration of different proposed modulation concepts. (A) Mechanical FSK. (B) Mechanical Shutter. (C) Electromagnet. (D) Electrically Modulated Reluctance (EMR).

**Mechanical Frequency Modulation:** This modulation concept is illustrated in Fig 2(A). This method suffers from low bandwidth due to the mechanical inertia of the magnet. In addition, even the most modest bit rates can result in low efficiencies. This can be shown by considering the modulation power needed to produce the same magnetic moment and bit rate demonstrated in [19]. Assuming a spherical magnet of the same size given in Table 1, the kinetic energy of the spinning magnet at a given frequency can be calculated. In [19], FSK modulation with a bandwidth of 2.5 Hz is used. We calculated (using standard textbook formulas for the kinetic energy of a rotating mass) 1.5 J of energy is needed in order to shift the frequency of the rotational magnet from 573.75 Hz to 576.25 Hz [20]. Given a very modest bit rate of 2 bits/sec, the modulation power for mechanical frequency modulation would be 3 W. In comparison, the total power consumption of the 2 m loop in [19] is 0.4 W. Due to high power consumption involved with mechanical modulation, attempts have been made to decrease the mechanical modulation power by using an array of smaller magnets [14]. However, a rigorous analysis of the effectiveness of this approach has yet to be demonstrated. There are potential disadvantages with this approach as well. Using a magnet array introduces greater complexity because the magnets must be synchronized. Furthermore, the volume of the transmitter for a given magnetic moment will be larger than using a single magnet due to spacing in the magnet array.

**Mechanical Shutter:** A magnetic shielding could be used as a type of shutter for amplitude modulation. The shutter would be made from a magnetically permeable material for shielding low frequency fields such as MuMETAL^®^, Permalloy 80, or Supermalloy. Depending on the mechanical design of the system, the shielding would rotate or vibrate. If the shield can be made to have much less moment of inertia than the spinning magnet, greater bandwidth and efficiency than mechanical FM modulation is possible. A possible design is illustrated in Fig 2(B). By spinning the shield in or out of phase with the magnet, the fields from the magnet could be modulated.

**Electromagnet:** An AC current carrying solenoid can be used to amplitude modulate the carrier from the spinning permanent magnet. This is illustrated in Fig 2(C). This method may produce much greater bandwidth than a mechanical modulation method can. However, the bandwidth should be the same as that of a conventional MI transmitter. The electromagnet could be phase or amplitude modulated. The fields from the electromagnet only need to modulate the carrier. However, if large modulation depth is desired, the efficiency of the system essentially becomes no different than that of a conventional MI transmitter.

**Electrically Modulated Reluctance (EMR):** We propose that a current carrying coil can be used to modulate the permeability of a shielding material (that encloses the magnet) and therefore the fields from the rotating magnet. The shield is to be driven in and out of magnetic saturation to act as a “shutter” for the spinning magnet. Our concept is illustrated in Fig 2(D). A cylindrical shielding surrounds the spinning magnet. This shield should be made from a highly permeable, low saturation flux density, low loss material such as MuMETAL^®^, Permalloy 80, or Supermalloy. A toroidal coil is wound around the shielding. By changing the current through the coil, the permeability of this shield changes in accordance with the B-H curve of the shielding material. A change in the permeability changes the effectiveness of the material to shield the fields of the spinning magnet.

In order to evaluate the efficiency of this approach, core losses in the shielding material and losses in the coil windings must be considered. The coil winding loss can be made low by using a large volume of copper. We illustrate this more fully with our prototype. Core loss due to eddy currents and hysteresis loss can be estimated with the Steinmetz’s equation [21]:

$$\begin{array}{c} P_{v}=Kf^{m}B^{n} \end{array}$$ (5)

Where *P*_*v*_ is the volumetric power density in [W/kg], *f* is the frequency of the magnetic field in [Hz], *B* is the magnitude of the field in [T], and *K*, *m*, *n* are constants that characterize the material. Using Eq 5, and known material constants for permalloy 80 (*K* = 2.41 × 10^−4^, *m* = 1.54, and *n* = 1.99) [21], the core loss density can be calculated. Assuming *B* = 0.75 T (the saturation flux density of Permalloy 80), we calculated a core loss of 1.95 W/kg at 500 Hz. In the Experimental Results section, we show that modulation of the magnet can be done with a very thin (0.254 mm thick) MuMETAL^®^ shielding (with similar properties to Permalloy 80). The total mass of our shield is only 42 g, which results in a small core loss of 80 mW at 500 Hz. Because core losses are low, the EMR approach can be designed to have high efficiency by using a large volume of copper for the toroidal windings.

In addition, we suggest that the bandwidth of this method is greater than that of a conventional MI transmitter. Conventional MI transmitters use a capacitor to form a resonant circuit with the transmitting coil. However, the resonant transmitter will have a finite bandwidth, producing limits on the maximum bit rate that can be transmitted. However, for EMR, the electrical excitation of the coils only contains the low frequency digital message signal. If the turn-on and turn-off time of the current through the coil is very small (much smaller than the carrier frequency), then very large fractional bandwidths are possible. In the Experimental Results section, we demonstrate a large fractional bandwidth of approximately 40%.

A summary of our comparison is shown in Table 2. Because the EMR modulation technique has the potential for giving the best performance in bandwidth and efficiency, we built a prototype that demonstrates this modulation technique.

**Table 2:** Qualitative comparison of each modulation technique.

Modulation Method	Power Efficiency	Bandwidth
Mechanical FM	Poor	Poor
Mechanical Shutter	Fair	Fair
Electromagnet	Fair	Good
EMR	Good	Excellent

## Experimental and simulation results

### Prototype design

**Fig 3:**
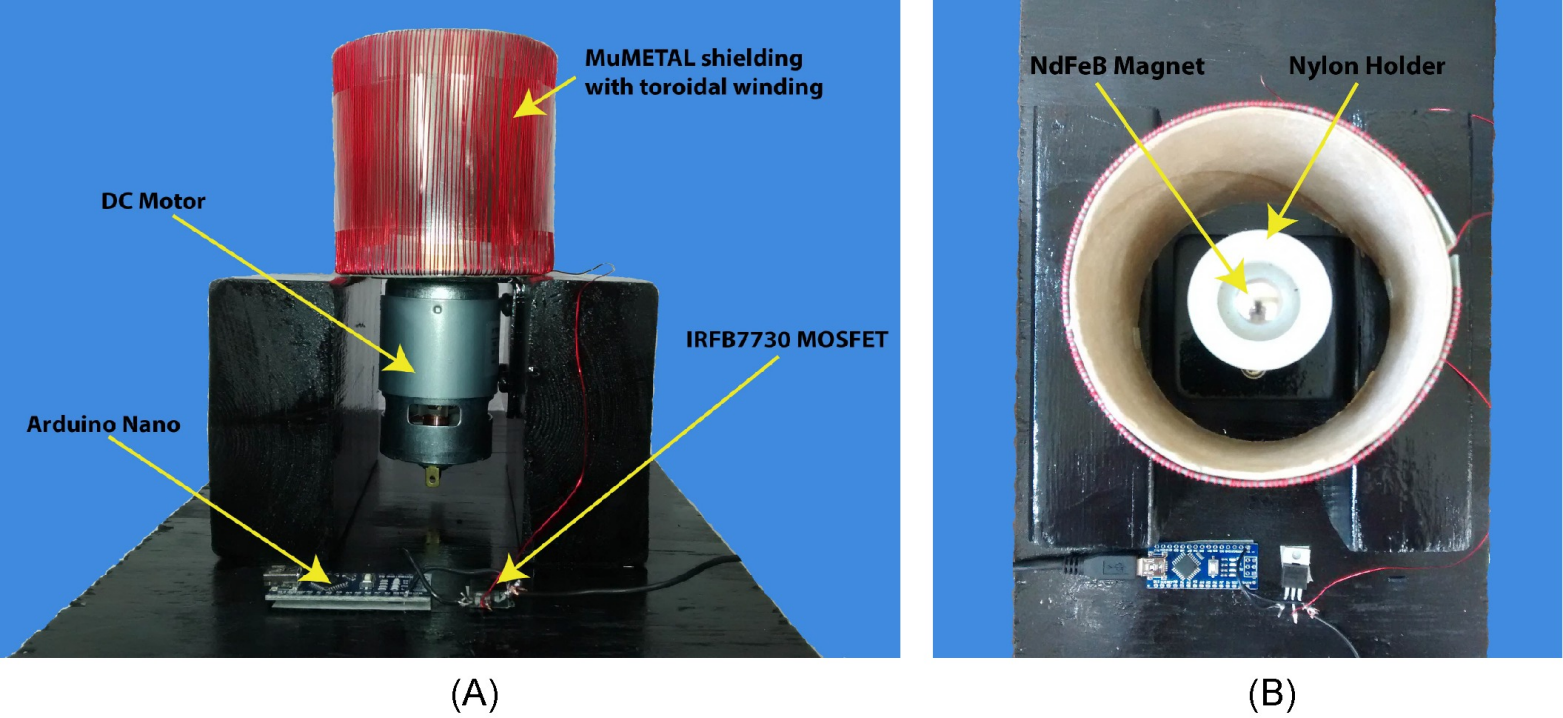
Construction of prototype. Mechanical MI transmitter prototype with electrically modulated reluctance (EMR). (A) Side view. (B) Top view.

We designed a simple prototype for proof of concept, shown in Fig 3. A motor capable of 12000 rpm (BEMONOC 775-12v12000rpm), was used to rotate a 1.905 cm diameter N42 spherical neodymium magnet (K&J Magnetics SC). A nylon cup with set screws was designed and machined to hold the magnet. To demonstrate EMR ASK modulation, an 8 × 7.5 cm [*L* × *D*] cylinder was constructed with 0.254 mm thick MuMETAL^®^ (LK-110, Fully Annealed), which is a high permeability material used for shielding low frequency fields. A toroidal coil of approximately 190 turns of 26 AWG wire was wrapped around the cylinder. The specific coil design was chosen for ease of prototyping. By changing the current through the coil the permeability of the MuMETAL^®^ changes and therefore the flux shunting capability of the shield. For proof of concept and convenience, the specific dimensions for the cylinder were chosen empirically, with no analytical derivations or design work.

**Fig 4:**
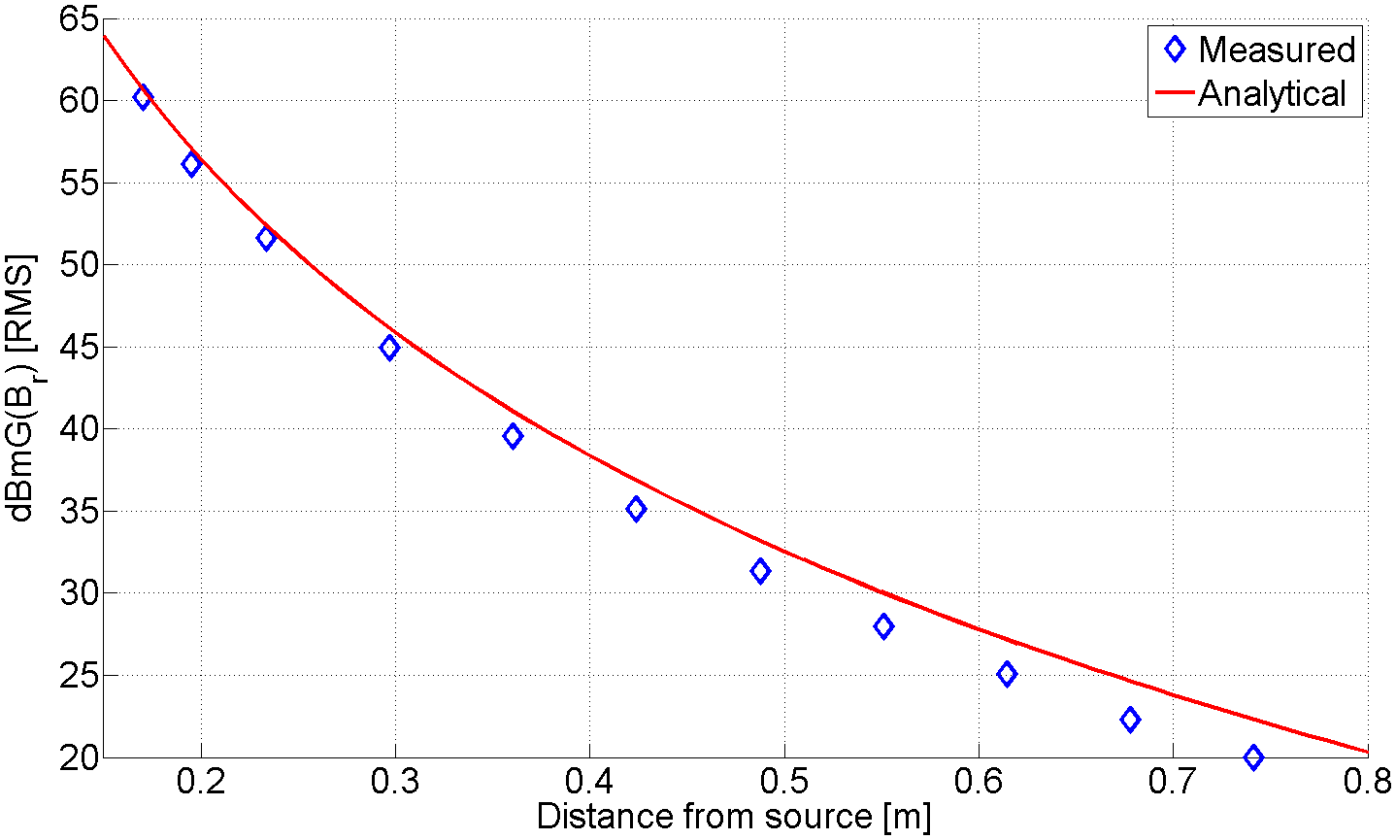
Received field strength. Measured radial magnetic field strength compared to analytically determined field strength. The magnetic field was computed based on the magnetic moment given by (3). We plot the fields in [dBmG] (*dBmG*(*B*_*r*_) = 20log(*B*_*r*_/1*mG*)) for easy comparison between experimental and theoretical results.

Without the shielding, the magnetic field from the spinning magnet was measured with a calibrated milli-Gauss meter (Integrity Design & Research Corp. IDR-210). In Fig 4, we plot the measured data points of the magnitude of the radial magnetic field and the field that is expected from (3) as a function of distance. Measurements were done in the plane orthogonal to the angular momentum of the magnet. Close agreement between theoretical and experimental results is demonstrated. The reason that the theoretical values are larger than the measured values is attributed to the fact that the receiver probe measures the field magnitude over an area (Approximately 12 *cm*^2^), rather than an exact point.

**Fig 5:**
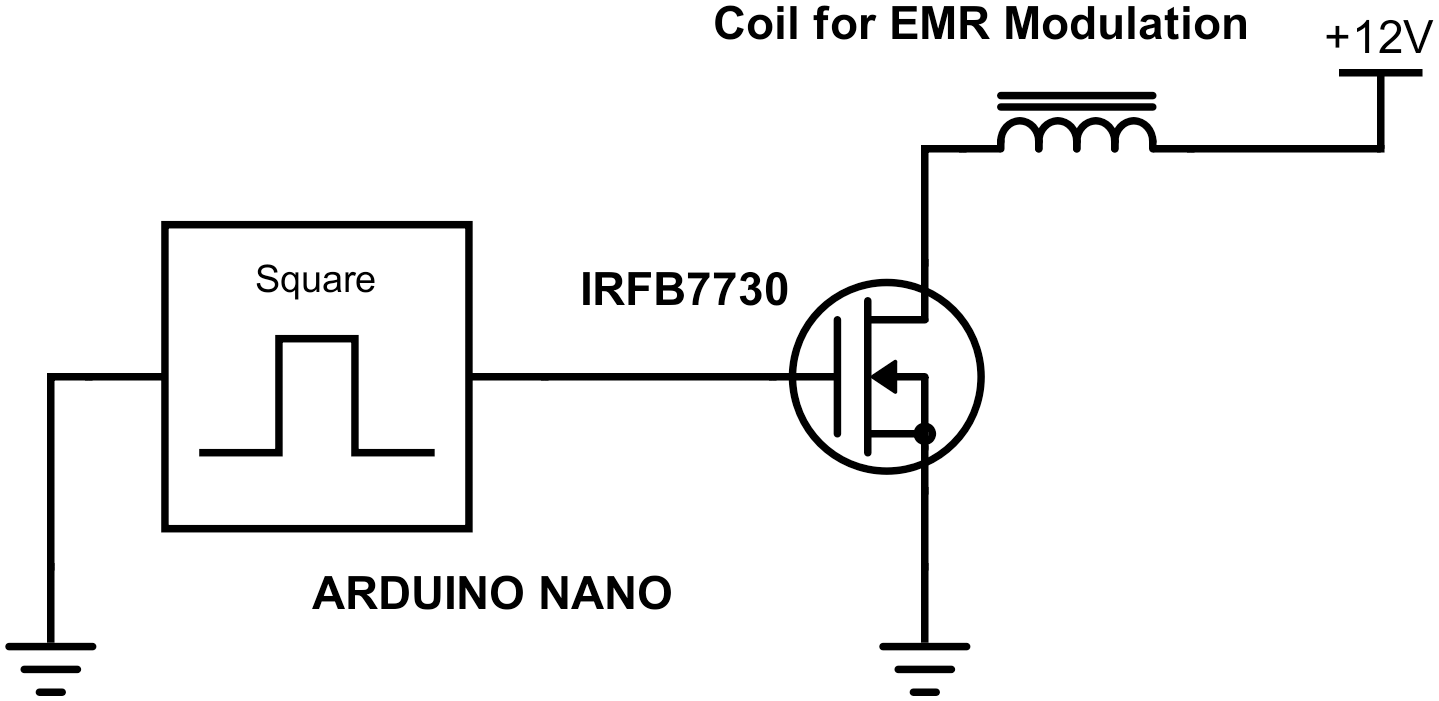
Switching circuit for ASK modulation. The square wave was produced by an Arduino Nano microcontroller.

**Fig 6:**
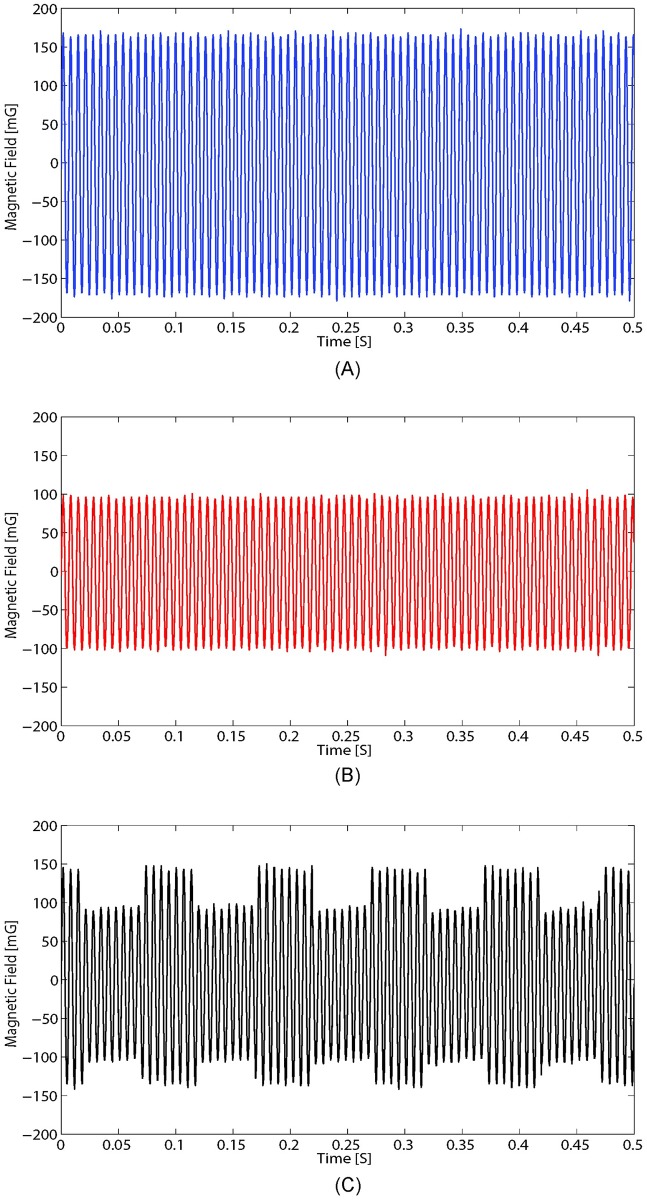
Measured 150 Hz waveforms from prototype. (A) Measured field with no shielding over the magnet. (B) Measured field with shielding placed over the magnet. (C) Received ASK modulated signal. Current through the coil was approximately 2.8 A, and the fields were measured a distance of 34.3 cm away from the transmitter.

To demonstrate modulation, the cylindrical shielding was placed over the spinning magnet, and the current through the toroidal coil was switched on and off with an IRFB7730 switching MOSFET. The MOSFET was controlled with an Arduino Nano microcontroller. The circuit schematic is shown in Fig 5. The analog output of the IDR-210 Milli-Gauss meter was connected to an oscilloscope, where the ASK modulated signal and non-modulated waveforms can be viewed in Fig 6.

### Magnetostatic simulation

**Fig 7:**
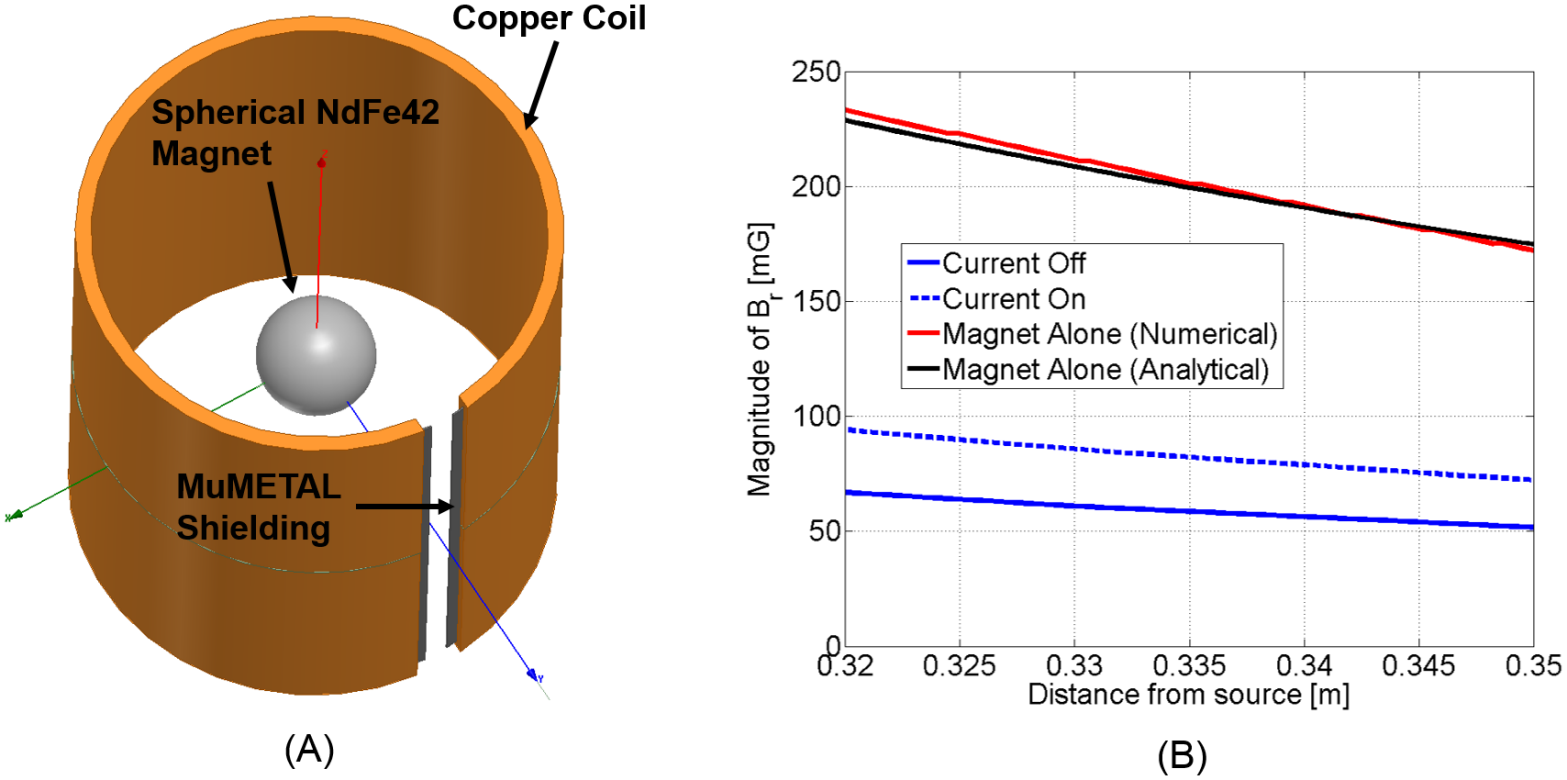
Simulation results: Prototype. (A) Magnetostatic simulation model. (B) Simulation results. The magnitude of the radial magnetic field facing the north pole of the magnet is plotted.

Subsequently, the behavior of the shield was modeled in ANSYS Maxwell, as shown in Fig 7. We used the magnetostatic simulator because of the low frequencies at which we were operating at. The far field strength of the field was simulated for three different cases: 1) Coil current on, 2) Coil current off, and 3) The magnet alone. The simulated field strengths for all three cases are shown in Fig 7(B). As can be observed, our simulations predict a modulation depth of a little over 30%, which agrees very well with our experimental results. However, the field strength with the shield over the magnet is predicted to be less than what was experimentally measured. We attribute this to differences in the B-H response of the experiment’s shield material and the ANSYS library B-H response model that we used in the simulation.

### Bandwidth

The bandwidth of our transmitter is determined by the rate at which current can switch through the coils, and by the carrier frequency. The switching speed is determined by the LR time constant of the coil, as well as the design of the switching circuit. In our particular prototype, the on and off current switching time was measured to be 2, and 28 *μs* respectively. This switching period is much faster than any kind of mechanical modulation. Since the switching speed is much faster than the period of the carrier frequency, the carrier frequency becomes the limiting factor in the bandwidth of our device. If higher data transmission rates are desired in our device, higher carrier frequencies must be produced. This involves spinning the magnet faster, which can become mechanically challenging.

**Fig 8:**
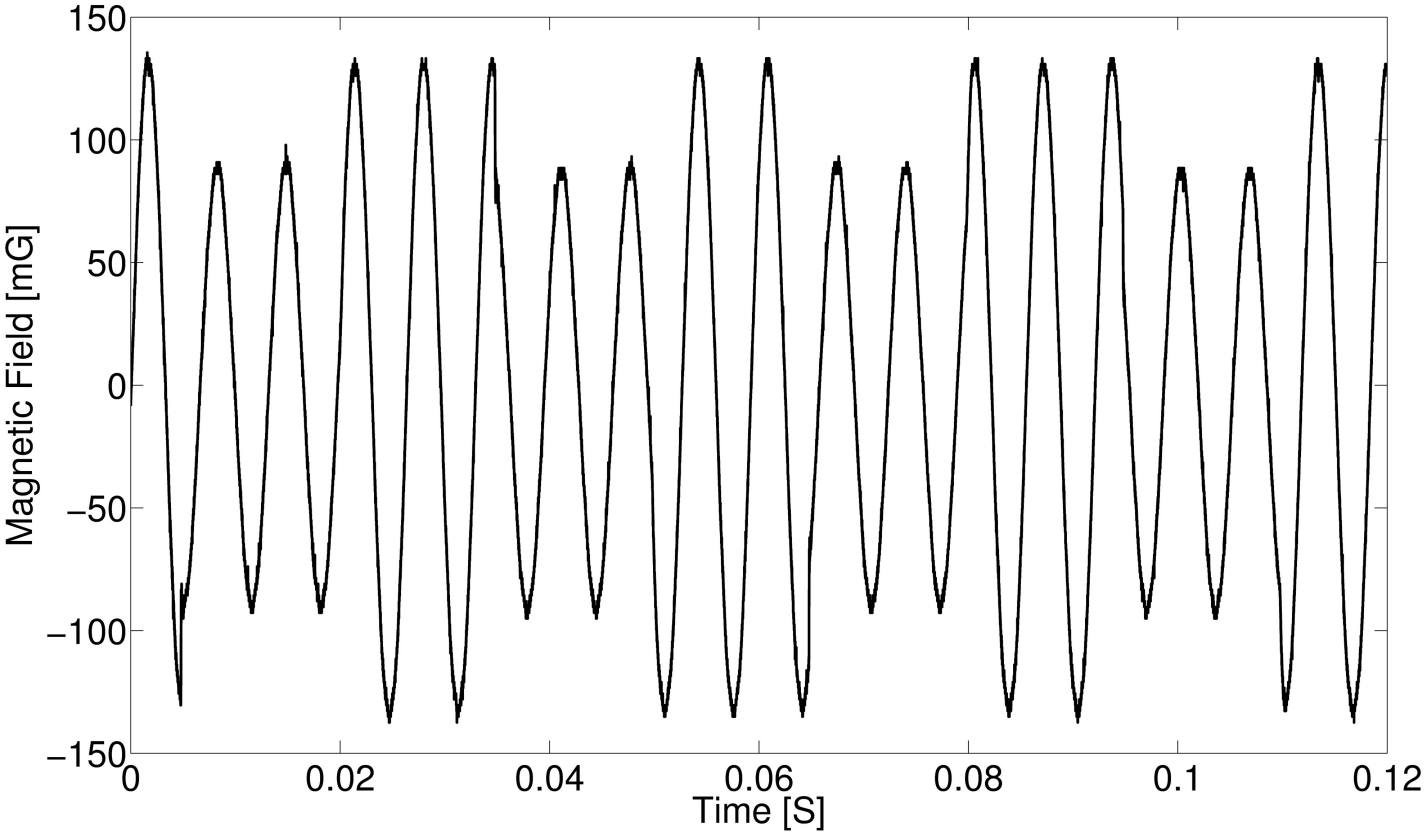
Wideband ASK modulation. A large fractional bandwidth of approximately 40% is shown. Current through the coil was approximately 2.8 A, and the fields were measured a distance of 34.3 cm away from the transmitter.

Another important factor to consider is that often the bandwidth of the communication system is dependent on the bandwidth of the receiver. A narrowband receiver is often used in order to have a high signal-to-noise ratio (SNR) [18]. However, we can conclude that for a given carrier frequency that is realistically achievable with a spinning magnet, the bandwidth of an EMR modulated mechanical transmitter surpasses that of a conventional MI transmitter. Since the switching speed is much faster than the period of the carrier, bandwidths that are a significant fraction of the carrier frequency are possible. In Fig 8, we demonstrate a data rate of 60 bits/sec (switching frequency of 30 Hz), yielding a large fractional bandwidth of approximately 40% (2 × 30/150 = 40%). It should be noted that we can produce larger fractional bandwidths with our prototype (up to 100%). A lower fractional bandwidth was shown to clearly view the modulation.

### Efficiency

The coil needed 2.8 A at 12 V to saturate the shielding. This corresponds to an average coil power consumption of 17 W (given a 50 percent duty cycle for the MOSFET). For ease of prototyping, we chose a small magnet and a low volume of copper for the windings. Because of this, the efficiency of our prototype is low. However, on the basis of our experimental results, high efficiency can be obtained using the EMR approach. In particular, efficiencies and bandwidths greater than what were reported in [19] are possible as explained in the following paragraphs.

**Fig 9:**
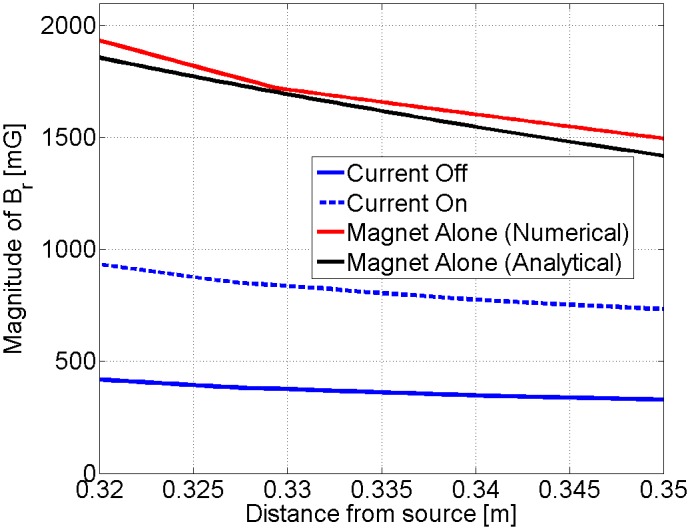
Simulation results: Larger magnet. The magnitude of the radial magnetic field facing the north pole of the magnet is plotted. We assumed the same number of ampere-turns of the coil as our original prototype.

In order to produce a magnetic moment of 30 *Am*^2^ (same as [19]), a spherical magnet that is twice the diameter of the one we used in our prototype is needed. If the magnet is made larger, the shielding should also be made larger to avoid saturation of the shield. We validated the performance of a shield that was twice the size of the prototype (scaled by a factor of two) by doing an additional ANSYS simulation to determine the modulation depth. As can be seen from Fig 9, a modulation depth of 44% is shown.

Due to low core loss in the shielding, ohmic losses in the windings are the main source of loss in our design. This means that using a large volume of copper will dramatically increase the efficiency of the transmitter. For example, we calculated (using experimentally verified formulas [22]) that using a multi-layer coil on our prototype wound from 1,000 turns of 28/20 (20 strands of 28 AWG wire) litz wire would reduce the power consumption from 17 W to 0.25 W. For a MuMETAL^®^ shield that is twice the size, a multi-layer coil wound from 1000 turns of 28/40 litz wire would have a power consumption of 0.25 W.

With a high speed motor, the carrier frequency could easily be scaled to 575 Hz. We suggest that a high-speed brushless DC motor such as the Portescap 16ECP36 ULTRA EC^™^ might be used. The no-load power consumption of the motor at 31,550 rpm is 1.92 W according to the data sheet [23]. We therefore assume that the power consumption for spinning the magnet at 34,500 rpm (575 Hz), would be approximately 2 W. It should also be noted that it may be possible to design a motor with higher efficiency, and ultra low friction bearings that could reduce the power consumption to less than 2 W. The power consumption of the coils would be nearly the same as the 150 Hz case because the coil only contains the low frequency message signal. Furthermore, as we previously calculated, the core loss for a sinusoidal signal at 500 Hz (with a magnitude great enough to saturate the shielding), yields a core loss of only 80 mW.

We previously showed that in order to mimic the results found in [19] with mechanical FSK modulation, a modulation power of 3 W for only 2 bits/sec would be required. It was argued from our experimental results that a modulation rate of 60 bits/sec with a power consumption of only 0.25 W (assuming the re-designed coil and larger magnet for a proper comparison) is possible with EMR. It should also be noted that the total power consumption reported in [19] for the 2 m diameter loop is 0.4 W. In addition, 60 bits/sec is also considerably more than the 2 bits/sec reported rate in [19]. Our results suggest that an EMR modulated spinning magnet can produce a greater efficiency and bandwidth in a much smaller volume than a conventional MI transmitter. We summarize our comparison in Table 3.

**Table 3:** Comparison of different MI transmitters. Mechanical FSK modulation was calculated assuming a spherical neodymium magnet and a fractional bandwidth of 0.40% (same as [19]). The carrier frequency, magnetic moment, power, efficiency, and maximum linear dimension are represented by *f*_*c*_, *m*_0_, *P*_0_, *e*_0_, and *l*_0_ respectively. Findings suggest that the EMR approach provides the best efficiency, bandwidth, and compactness for larger magnetic moments.

Transmitter	*f*_*c*_ [Hz]	Mod. Rate [bits/sec]	*m*_0_[*Am*^2^]	*P*_0_ [*W*]	*e*_0_ [*Am*^2^/*W*]	*l*_0_ [m]
Dinn [19]	575	2	30	0.4	75	2.0
Mechanical FSK	575	2	30	3.0	10	0.037*
EMR Prototype Demonstrated	150	60	3.8	17	0.22**	0.11
Mechanical FSK	150	60	3.8	0.19	20	0.018*
EMR Prototype, scaled	575	60	30	0.25 (2.25)**	120 (13.3)**	0.22
Mechanical FSK	575	60	30	89	0.34	0.037*

*Maximum linear dimension does not include size of the motor.

**Including estimated motor power consumption of 2 W.

Besides improving the coil design and magnet size, there are several other designs that could potentially increase the efficiency of our transmitter:

**Permanent Magnet Bias:** The efficiency of EMR modulation may be increased by using a static permanent magnet to bias the shield at an optimum point on the B-H curve of the shielding material. If the shield is biased at a point on the B-H curve just before saturation, then less current will be needed in order to “push” the shield into saturation.

**Low Modulation Depth:** In our prototype, we produced a modulation depth of 33%. However, lower modulation depths may be sufficient. We observed experimentally that changing the current through the coil by 0.5 A produced a modulation depth of 11% in the received field. This modulation depth may be large enough for communication purposes. This would reduce the average modulation power consumption from 17 W to 0.54 W in our current design. Using this technique in conjunction with using thicker wires for the modulating coil can further decrease the power consumption of the modulation coil.

**Composite Materials:** Instead of using a single type of shielding material, it may be possible to build a shield that is comprised of different sectors or layers of material. An optimum shielding material could be synthesized.

## Discussion

Mechanically based magneto-inductive transmitters are a promising technology for MI communications. Much work remains to be done in evaluating the performance characteristics of mechanically based MI transmitters. We have demonstrated that it is possible to generate a strong magnetic moment in a small volume with a neodymium magnet. Very low power consumption is possible with efficient motors and low friction bearings. A wideband ASK modulation technique was proposed and demonstrated. Results also indicate that high efficiency modulation is possible. Challenges in realizing a mechanical MI transmitter are mechanical stability, efficient mechanical rotation, and efficient modulation. Using a single magnet, the mechanical spinning frequency determines the carrier frequency. Applications requiring large magnets may create challenges for scaling to high carrier frequencies. However, this challenge may be addressed by using a multi-pole rotor instead of a dipole magnet. Work also remains to be done with modeling the shield that encloses the magnet based on physical dimensions and material properties. This will allow a quantitative analysis on the efficiency of EMR modulation. Additionally, in order to optimize the bandwidth of the device a proper switching circuit must be designed for ASK modulation.
